# Mechanisms of Macrophage Immunomodulatory Activity Induced by a New Polysaccharide Isolated From *Polyporus umbellatus* (Pers.) Fries

**DOI:** 10.3389/fchem.2020.00581

**Published:** 2020-07-22

**Authors:** Chun-Ping Liu, Xiong Li, Ge-Na Lai, Jin-Hua Li, Wen-Yu Jia, Ying-Ying Cao, Wen-Xing Xu, Qing-Long Tan, Chang-Yuan Zhou, Min Luo, Xue-Ying Zhang, Dao-Qing Yuan, Jin-Ying Tian, Xian Zhang, Xing Zeng

**Affiliations:** ^1^Department of Integrated Chinese Medicine Immunization, The Second Affiliated Hospital, Guangzhou University of Chinese Medicine, Guangzhou, China; ^2^Department of Cardiovascular Medicine, Guangdong Provincial Hospital of Chinese Medicine, Guangzhou, China

**Keywords:** *Polyporus umbellatus*, polysaccharide, immunomodulatory activity, NLR P3 inflammasome, NF-κB pathway

## Abstract

Bladder cancer is one of the most malignant tumors closely associated with macrophage immune dysfunction. The Chinese medicine polyporus has shown excellent efficacy in treating bladder cancer, with minimal side effects. However, its material basis and mechanism of action remain unclear. A new water-soluble polysaccharide (HPP) with strong immunomodulatory activity was isolated from the fungus *Polyporus umbellatus* (Pers.) Fries. HPP had an average molecular weight of 6.88 kDa and was composed mainly of an <-(1 → 4)-linked D-galactan backbone. The immunomodulatory activity of HPP was determined *in vitro*, and the results revealed that it could obviously increase the secretion of immune factors by IFN-γ-stimulated macrophages, including nitric oxide (NO), interleukin-6 (IL-6), interleukin-1β (IL-1β), RANTES and interleukin-23 (IL-23), and the expression of the cell membrane molecule CD80. In addition, HPP was recognized by Toll-like receptor 2 (TLR2) and activated the signaling pathways of NF-κB and NLRP3 in a bladder cancer microenvironment model, indicating that HPP could enhance host immune system function. These findings demonstrated that HPP may be a potential immune modulator in the treatment of immunological diseases or bladder cancer therapy.

## Introduction

Bladder cancer, the most common malignancy affecting the urinary tract, is characterized by the proliferation of abnormal cells in the urothelial lining of the urinary bladder (Zhang et al., 2012; John and Said, 2017; Kolawole et al., 2017; Moschini et al., 2017). According to statistics, more than 100,000 people die from bladder cancer every year worldwide, ranking fourth in incidence among male malignant tumors (Ferlay et al., 2015; Dy et al., 2016). The traditional treatment of bladder cancer is mainly surgery combined with BCG intravesical perfusion. Although BCG has a good healing effect, it is also accompanied by serious side effects, and at the same time, since as many as 30–50% of patients' relapse within 5 years, clinical doctors have to find other auxiliary therapies (Kawai et al., 2013).

Macrophages are important immune cells with great functional diversity and a series of functions, including immunomodulation of host defense, resolution of inflammation, and maintenance of various homeostatic processes (Saha et al., 2017). To carry out these seemingly contrasting functions, macrophages need to display functional plasticity in response to micro-environmental signals (Solinas et al., 2010). Clinical and experimental data indicated that bladder tumor-related macrophages ware heterogeneous and can be induced to present tumor-associated macrophages (TAMs) that are similar to the M2 subtype in the tumor microenvironment, promoting tumor growth, invasion, invasion and angiogenesis (Suriano et al., 2013; Qi et al., 2019). Generally, TAMs promote tumor growth and angiogenesis, suppressing adaptive immunity, and play important roles in tumor cell migration, invasion, and metastasis (Wu et al., 2020). M1 subtype macrophages differentiate through mainly JAK1/2, STAT1/2, 5, TLR4/NF-κB, P38 MAPK pathway activation, excrete inflammatory cytokines and chemokines, participate in a positive immune response; as such, they are able to kill tumor cells in tumor tissue and foreign pathogens. The immune function of M1 macrophages is achieved mainly by secreting cytokines, such as IL-6, TNF-α, IL-1β, IL-23 and other inflammatory factors, as well as chemokines, such as McP-1, RANTES and CXCL10 (Gambardella et al., 2020). Interferon- γ (IFN-γ) is a major macrophage activation factor responsible for M1 macrophage activation. Compared with unstimulated RAW 264.7 cells treated with T24 cell culture supernatant, IFN-stimulated macrophages are characterized by the production of increased quantities of inflammatory cytokines in medium alone or in a co-culture microenvironment. IFN-stimulated macrophages are implicated in acute inflammation and defense against tumors. Therefore, in this study, the treatment of RAW 264.7 cells with T24 cell culture supernatant was used as a model to simulate the bladder tumor microenvironment *in vitro*, and the macrophages were activated by exposure to IFN-γ (Liu et al., 2017).

*Polyporus umbellatus* is a Chinese traditional herbal medicine, also known as Zhuling in China, that shows diuretic, nephron-protective, anticancer, immune-stimulating, hepatoprotective, anti-inflammatory and anti-oxidative activities (He P. F. et al., 2016). Clinical applications have confirmed its satisfactory efficacy in treating kidney diseases and bladder cancer. Additionally, no side effects or toxicity have been reported for this fungus (Zhao, 2013). Polyporus polysaccharide, which is the main ingredient in *Polyporus umbellatus*, has multiple pharmacological functions, including anticancer, immunoenhancing, radioprotective and antioxidative activities (He P. F. et al., 2016). In a previous study, we found that crude polyporus polysaccharide could suppress the growth of tumor cells via the TLR4/NF-κB pathway (Zeng et al., 2011) and greatly induce the transformation of M2 subtype macrophages to the M1 subtype *in vitro* (Jiang et al., 2015). In addition, we measured the toxicity of PPS in a rat model of bladder cancer *in vivo* and found that the mortality of the Bacillus Calmette-Guérin (BCG)-treated group was higher than that of the group treated with PPS combined with BCG (Zhang et al., 2010, 2011), indicating that PPS can reduce the toxicity of BCG.

In this study, a new polysaccharide (HPP) was first isolated from polyporus total polysaccharide, which was proven to have an α-(1 → 4)-linked D-galactan backbone. We further examined whether HPP could regulate macrophage polarization in the microenvironment of bladder cancer and thereby exert anticancer effects through the NF-κB and NLRP3 pathways.

## Materials and Methods

### Antibodies and Reagents

RAW 264.7 macrophages and T24 cells were purchased from the ATCC (Rockville, MD, USA). Antibodies for western blotting were purchased from Cell Signaling Technologies (Pickering, ON, CAN), including those targeting P65, P-P65, TAK1, iKKa/b, INOS, COX2, IKB, p-IKB, NLRP3, Caspase-1, and GAPDH. Secondary antibodies for western blotting and 3-(4,5-dimethylthiazol-2-yl)-2,5- diphenyltetrazolium bromide (MTT) were purchased from Sigma (Sigma Aldrich, St. Louis, MO, USA). Transitional cell carcinoma of the bladder cells (T24 cells) and a mouse macrophage cell line (RAW 264.7 macrophages) were purchased from the American Type Culture Collection (Rockville, MD, USA). Fetal bovine serum (FBS), Dulbecco's modified Eagle's medium (DMEM) and penicillin/streptomycin solution were purchased from HyClone (Logan, UT, USA). Phycoerythrin (PE)-conjugated anti-CD80 and fluorescein isothiocyanate (FITC)-conjugated anti-mouse CD282, PE-IgG2a and FITC-IgG2a antibodies, were purchased from BD Systems (BD Biosciences, USA). ProcartaPlex^TM^ Multiplex Immunoassay Kits were purchased from eBioscience (San Diego, CA, USA). DEAE-52 and Sephadex G-100 gel filtration medium were purchased from GE Healthcare Bio-Sciences AB (UPPPala, Sweden). SC75741 (purity, 99.79%) was purchased from Selleck (Shanghai, China).

### Isolation and Purification of Polysaccharides

Total *Polyporus umbellatus* polysaccharide was purchased from Hui Zhou Xian Cao Plant Health Care SCI & THE Co., Ltd. (Huizhou, China, batch number 150780681), and the polysaccharide content determined by the phenol-sulfuric acid method was 73.5%. The polysaccharides were deproteinized using Sevag reagent (1-butanol/chloroform, v/v = 1:4), and the supernatant was lyophilized to obtain the deproteinized polysaccharides. The deproteinized polysaccharides were dissolved in deionized water, after which the solution was applied to a DEAE-52 cellulose column (3 × 35 cm) and eluted with deionized water at a flow rate of 1 mL/min. Test tubes were collected using an automated step-by-step fraction collector, after which the total carbohydrate content of each tube was measured based on the absorbance at 490 nm using the phenol-sulfuric acid colorimetric method. The main fraction containing carbohydrates from the elution step was then concentrated and lyophilized. The collected fraction was further applied to a Sephadex G-100 gel-filtration column (2.9 × 50 cm), after which it was eluted with deionized water at a flow rate of 1.0 mL/min. The neutral carbohydrate was eluted as a single fraction according to the elution profile and lyophilized as a white powder.

### High-Performance Gel-Permeation Chromatographic Analysis

The homogeneity and molecular weight of the purified polysaccharide were determined using high-performance gel-permeation chromatography (HPGPC) via an Agilent-1,200 HPLC system matched with a TSK gel G4000 PWxl column (7.8 mm × 300 mm,), column temperature 30°C, and detected using a differential refraction index detector (RID) at 35°C. The sample was dissolved in distilled water to a concentration of 0.5 mg/mL and then eluted at a flow rate of 0.6 mL/min. A calibration curve was constructed using dextrans of various molecular weights (Mw 4,300, 5,300, 7,200, 9,200, 16,230 and 17,900).

### UV, ORD, IR, and NMR Analysis

UV–Vis absorption spectra were recorded using a U-2,910 spectrophotometer (Hatachi, Japan) in the wavelength range of 200–400 nm. The FT-IR spectra (KBr pellets) of the polysaccharides (2 mg) were recorded at 400–4,000 cm^−1^ using a Fourier transform infrared spectrophotometer (FT-IR, PerkinElmer, UK) at room temperature. The optical rotatory dispersion (ORD) was recorded using a Rudolph I automatic polarimeter (Rudolph Research Analytical, USA). 1D and 2D NMR spectra were recorded using a Bruker 5 mm broadband observe probe at 20°C with a Bruker Avance 600 MHz spectrometer (Germany), operating at 600 MHz for ^1^ H NMR and 150 MHz for ^13^C NMR,™ in ppm, rel. to SiMe_4_ as an internal standard, *J* in Hz.

### Monosaccharide Composition Analysis

The monosaccharide composition of HPP was analyzed by GC-MS and PMP derivatization HPLC. For GC-MS analysis, 10 mg samples were hydrolyzed using 4 mol/L trifluoroacetic acid (TFA) at 120°C for 4 h, after which the TFA was removed using nitrogen at 25°C. Next, 10 mg of hydroxylamine hydrochloride and 0.6 mL of pyridine were added, reacted at 90°C for 0.5 h, evaporated with nitrogen and diluted in 2 mL of CHCl_3_. The reaction products were subsequently analyzed by gas chromatography–mass spectrometry (GC–MS, QP2010, Shimadzu, Japan) using a DB-1701 silica capillary column (30 m × 0.25 mm × 0.25m). During GC–MS, the flow rate of helium was 1 mL/min, the detector and inlet temperatures were 280 and 250°C, respectively, and the oven temperature program was set to increase from 100°C (standing for 2 min) to 260°C (standing for 15 min) at a rate of 15°C/min. For PMP derivatization HPLC, five milligrams of PPS were dissolved in NaOH and precolumn derivated with 1-phenyl-3-methyl-5-pyrazolone (PMP) using the method described by Ma et al. (2017). The PMP derivatives of the seven standard sugars (Ara, Gal, Glc, Gal UA, Man, Rha) and PPS were subjected to HPLC with an Agilent 1,200 (Dionex Co., USA) fitted with a HypersiL BDS C_18_ column (250 × 4.6 mm, 5 μm).

### Cell Line Culture and Tumor-Conditioned Media Preparation

RAW 264.7 macrophages and T24 cells were cultured in Dulbecco's modified Eagle's medium (DMEM) with 10% fetal bovine serum (FBS) containing 1% penicillin/streptomycin. For all experiments, cell lines were grown at 37°C in an atmosphere of 5% CO_2_. The T24 supernatant was collected and filtered at 0.20 μm, after which the supernatant was stored at −80°C. Once the cells were grown to 80% confluence, 40% of the medium was discarded, and the cells were incubated with fresh DMEM or T24 supernatant for 3 h. Finally, the samples were treated with different concentrations of IFN-γ (100 ng/mL) with or without HPP for 24 h, with equal volumes of medium used as controls.

### MTT Cell Viability Assay

RAW 264.7 cells (1 × 10^4^ cells/well) were seeded into 96-well microtiter plates for 24 h at 37°C with 5% CO_2_. Various concentrations of HPP (3.75–1,000 μg/mL) were then added and co-cultured for 24 h, after which 20 l of MTT solution (5 mg/mL) was added into each well, and samples were incubated at 37°C for 4 h. Following incubation, the supernatant was removed, and 200 μL of dimethyl sulfoxide was added to solubilize the formazan salt, which was then quantified based on the absorbance at 570 nm. The control wells were designated 100% viability, and the blank values, indicating the absorbance of MTT and DMSO only, were subtracted from all samples. Each experiment was repeated at least three times. The cell viability (%) after HPP treatment of RAW264.7 cells was calculated as follows: (absorbance of test sample/absorbance of control) ×100%.

### Measurement of NO Production

RAW 264.7 cells (5 × 10^5^ cells/well) were preincubated in six-well plates for 24 h at 37°C in a 5% CO_2_ incubator. The cells were preincubated with 40% T24 cell culture supernatant for 3 h before being treated with IFN-γ (100 ng/mL) or HPP (1–100 μg/mL) for 24 h. After treatments, the supernatant was harvested and measured based on the Griess reaction.

### Multiplex Immunoassay Analysis

RAW 264.7 cells (2.5 × 10^5^ cells/well) were seeded in 12-well plates and cultured for 24 h at 37°C in a humidified atmosphere containing 5% CO_2_. Cells were treated with 40% T24 cell culture supernatant for 3 h. Subsequently, the cells were stimulated with IFN-γ (100 ng/mL) or HPP (1–100 μg/mL) for 24 h. The supernatant was then harvested, and the levels of RANTES, IL-23, IL-1β and IL-6 in the RAW 264.7 culture supernatant were assayed using ProcartaPlex^TM^ Multiplex Immunoassay Kits according to the manufacturer's instructions.

### Flow Cytometry Analysis

The cell phenotypes were determined using a flow cytometer. RAW 264.7 macrophages (5 × 10^5^ cells per well) were plated in 12-well culture plates. After 24 h, the culture medium was replaced with T24 cell culture supernatant for 3 h before incubating with IFN-γ or with different concentrations of HPP (1–100 μg/mL) for 24 h. Finally, we harvested the treated cells and washed them twice with cold phosphate-buffered saline (PBS). Next, 5 μL of anti-mouse (PE)-conjugated anti-CD80, (FITC)-conjugated anti-mouse CD282, PE-IgG2a, and FITC-IgG2a antibodies, were added to the tubes and incubated for 20 min on ice. Finally, the stained cells were suspended in cold buffer, and fluorescence-activated cell sorting (FACS) was used to analyze the data.

### Western Blot Analysis

After various treatments, cells were washed with ice-cold phosphate buffer, lysed in 1× RIPA lysis buffer and then harvested. The lysates were subsequently centrifuged at 6,000 × g for 10 min at 4°C. Before analysis, the proteins in the supernatant were measured using a Thermo protein assay kit. Protein samples were loaded, separated on 10% gels and transferred to polyvinylidene difluoride membranes (Immun-Blot PVDF membrane, 0.45 μm). The membranes were then blocked with 5% BSA in 1× Tris-buffered saline (TBS) for 2 h, after which they were incubated with primary antibodies at 4°C overnight. The membranes were subsequently washed and incubated with horseradish peroxidase-conjugated secondary antibody raised against rabbit IgG for 1.5 h at room temperature. After washing with TBST, membranes were detected using an enhanced chemiluminescence (ECL) kit.

### Statistical Analysis

The experimental data were measured four times, and GraphPad Prism 6.0 was used for statistical analysis. Groups were compared using Student's *t*-test. Data are presented as the means ± SEM. *p* < 0.05 was considered statistically significant.

## Results and Discussion

### Structural Assignment of HPP

#### Determination of the Molecular Weight (M_w_) of HPP

**Figure 1:**
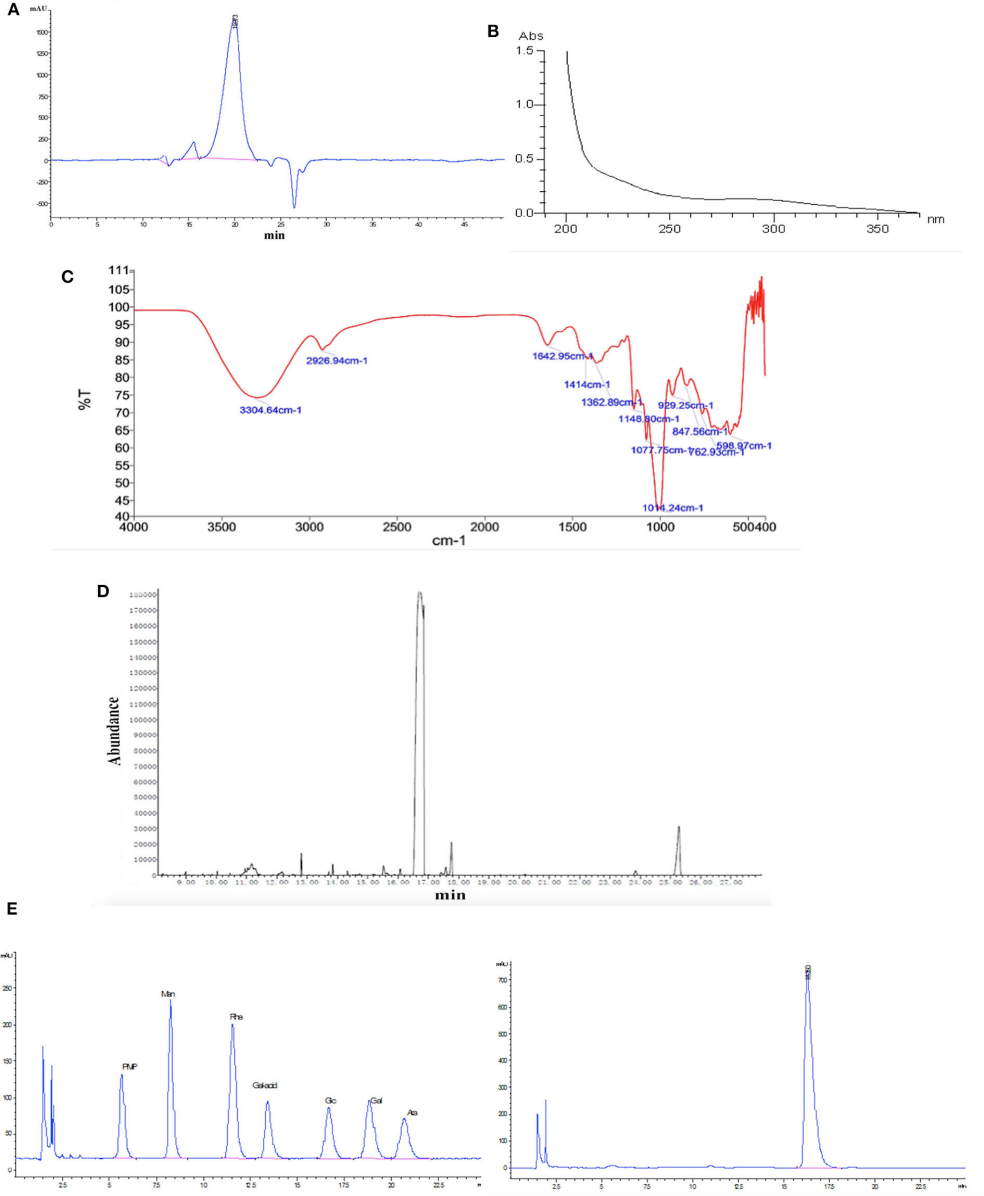
Characterization of HPP by **(A)** high-performance gel permeation chromatography (HPGPC), **(B)** UV scanning spectrum, **(C)** infrared spectrum, **(D)** gas chromatography of the glucose standard (16.6 min), and **(E)** gas chromatography of the HPP sample (16.5 min).

The purified polysaccharide appeared as a single and symmetrical sharp peak upon HPGPC analysis (Figure 1A), indicating that HPP was a homogeneous polysaccharide. The average molecular weight of HPP was determined to be 6.88 kDa.

#### UV, FT-IR, GC, and PMP Derivatization HPLC Analysis of HPP

The UV spectra revealed that there was no absorption at 260 or 280 nm, suggesting that nucleic acids and proteins were absent (Figure 1B; Wang et al., 2017). FT-IR spectroscopy showed that HPP had typical features of sugars (Figure 1C). A strong and broad absorption peak at 3,304.6 cm^−1^ corresponded to the O-H stretching vibration, while a weak C-H stretching vibration was observed at 2,926.9 cm^−1^. The absorption peaks between 1,400 and 1,200 cm^−1^ may be associated with O-H deformation vibration and C-O stretching vibration. The bands at 1,148.8, 1,077.8 and 1,014.2 cm^−1^ contributed to the stretching vibration of sugar structures with pyranose configurations. There was no absorption at 1,740 cm^−1^ or 890 cm^−1^ for the uronic acid and ™-type glycosidic linkages, respectively. Moreover, a characteristic absorption band at 847.6 cm^−1^ indicated the presence of <-type glycosidic linkages (Li et al., 2008). The high positive ORD values (${+158.4}_{,}^{{}^{\circ}}$ c, 1.0, H_2_O) further indicated that the polysaccharide is composed of <-D-glucose (Li et al., 2013). GC and PMP derivatization HPLC analysis showed that HPP was composed only of glucose (Figures 1D,E).

#### NMR Analysis of HPP

**Figure 2:**
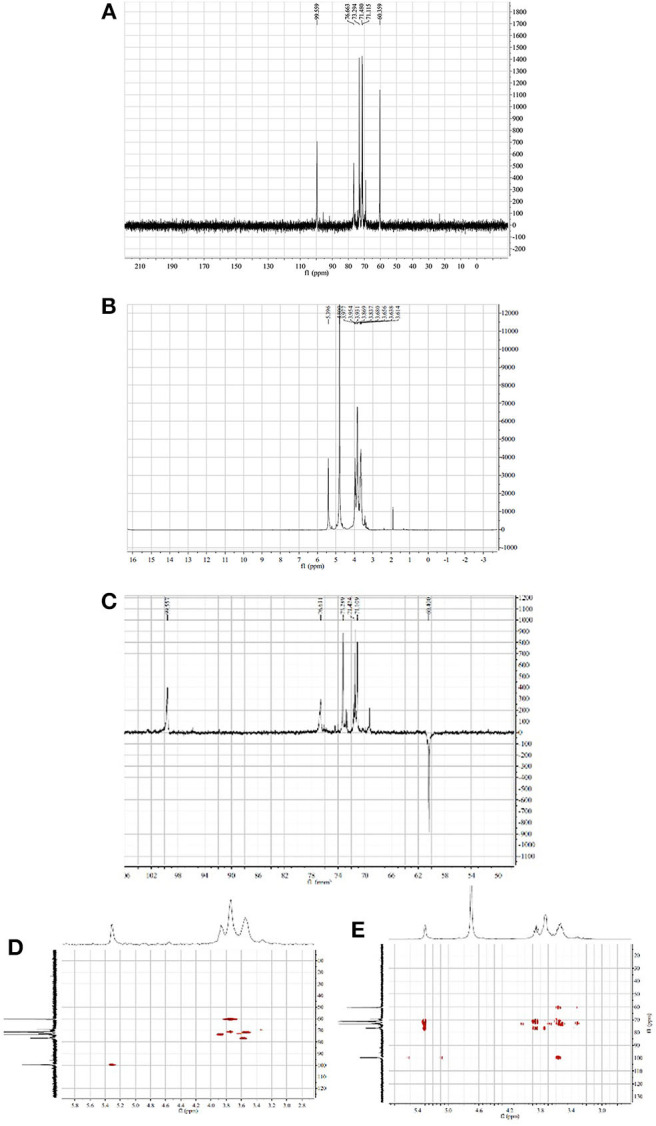
NMR spectra of HPP (solvent: D_2_O). **(A–E)** represent the ^1^H NMR spectrum, ^13^C NMR spectrum, DEPT spectrum, HSQC spectrum and HMBC spectrum at 60°C, respectively.

The ^1^H and ^13^C NMR spectra of HPP are shown in Figures 2A,B. The anomeric proton signals of residues were >5.0 (δ 5.40 ppm), and the broad single peak indicated that the glucosidic bond is connected to the alpha form. The rest of the hydrogen chemical shifts were in the range of 3.61–3.98, corresponding to other proton signals on the sugar ring carbon, and the values were <4.0, indicating that the polysaccharide has no other sugar residue anomeric hydrogen signal. The ^13^C-NMR spectrum showed six major carbon signals with chemical position values of 99.6, 76.6, 73.3, 71.5, 71.1, and 60.4. The anomeric carbon signal (δ 99.6 ppm) indicated that the sugar was in the pyranose form. The signal at δ 60.4 ppm was assigned to C-6 of (1 → 4)-α-D-glucosyl residues, which was supported by the corresponding reversed peak in the DEPT spectrum (Figure 2C). When combined with HSQC and HMBC correlation analysis (Figures 2D,E), the carbon and hydrogen signals were completely attributed to HPP, wherein δ 99.6 reflected the anomeric carbon signal of glucose residues; δ 71.5, 71.1 and 73.3 were the C-2, C-3, and C-5 carbon signals of glucose, respectively; δ 76.6 was the substituted C-4 resonance signal; and δ 60.4 was the C-6 carbon signal, and its monosaccharide composition contained only α-D-glucopyranose, which is consistent with the above GC-MS, and IR analysis results. The chemical shifts of C-1 (δ 99.6) and C-4 (δ 76.6) move to the lower field indicates that the hydroxyl groups at C-1 and C-4 are substituted, which was further confirmed by the remote correlations of H-1 (δ 5.40) /C-4 (δ 76.6), and H-4 (δ 3.61) / C-1 (δ 99.6) in the HMBC spectrum. Other carbon signals with chemical shift values of 99.7, 76.8, 72.8, 72.6, 71.6, and 69.3 were also detected in the carbon spectrum, where δ 69.3 is the substituted C-6 signal, indicating that there is branching at the O-6 position, and δ 99.7, 76.8, 72.8, 72.6, and 71.6 are the C1-5 carbon signals of 1,4,6-α-D-glucopyranose, but these carbon signal intensities were weak. The above analysis indicated that the homogenized polysaccharide HPP contains glucose alone, having a backbone of 1,4-linked α-D-glucan with a (1 → 6)-α-D-glucopyranosyl side-branching unit. To our knowledge, the polysaccharide HPP isolated from *Polyporus* sp. have not previously been reported and was tentatively identified as novel.

### Effects of HPP-Induced NO Production in IFN-γ-Stimulated Macrophages

**Figure 3:**
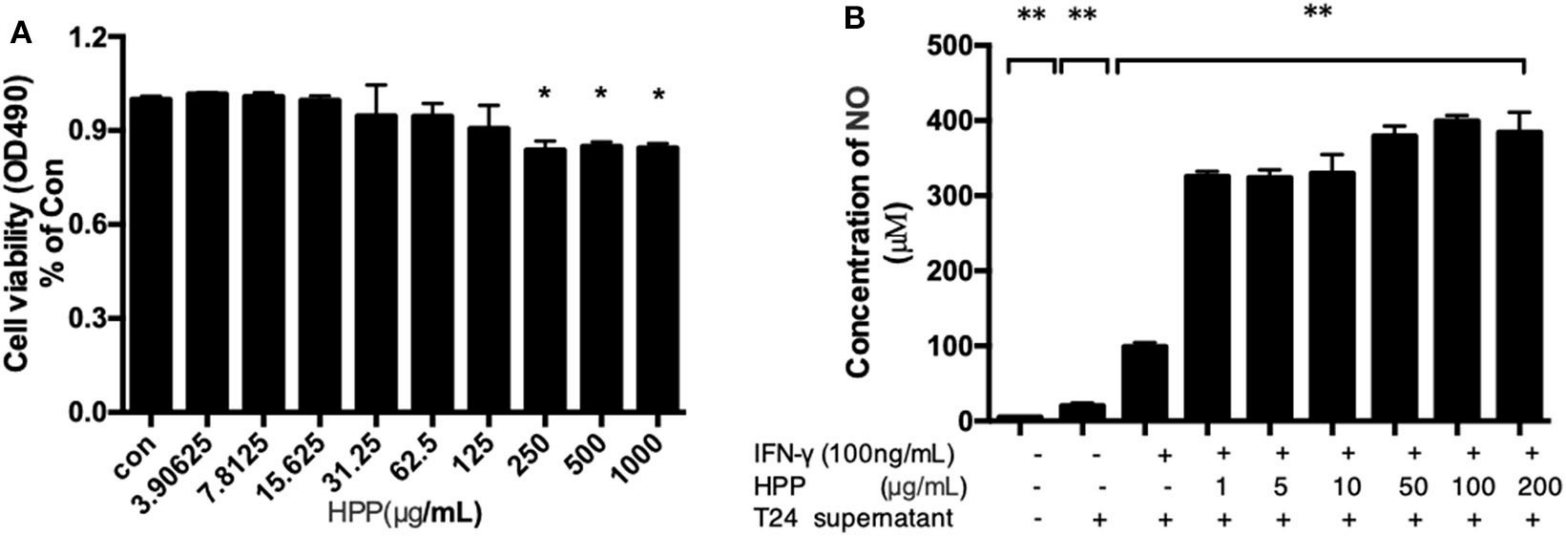
Effect of HPP on cell viability and secretion of NO in IFN-γ-induced RAW 264.7 cells. **(A)** Effect of HPP on cell viability. **(B)** Effect of HPP on NO production. Statistical significance in comparison to the control group is designated *P < 0.05, **P < 0.01. Data are presented as the mean ± s.e.m.

To investigate the effects of HPP on the viability of RAW 264.7 macrophages, cells were treated with different concentrations of HPP (3.9–1,000 μg/mL) for 24 h, after which the cell viability was determined by MTT assay. The results showed that HPP at 3.9–125 μg/mL did not affect cell viability (Figure 3A), which indicated that HPP at <125 μg/mL was not obviously toxic to RAW264.7 cells.

NO is an important active molecule in living organisms that plays pivotal roles in multiple pathophysiological processes, such as host defense mechanisms, inflammation, cancers and immunological diseases (Pratap et al., 2015). In this study, we found that treatment with HPP significantly induced the production of NO in IFN-γ-stimulated RAW264.7 cells in a dose-dependent manner. In particular, cells treated with HPP (1–1,000 μg/mL) showed significantly higher NO production than those in the control group (Figure 3B). When compared with that in the IFN-γ-stimulated RAW264.7 group, the amount of NO increased significantly after treatment with 1 μg/mL HPP (*P* < 0.01) until reaching 321 pg/mL, which is higher than that induced by rough polyporus polysaccharides (Liu et al., 2017).

### Increase in IFN-γ-Stimulated CD80 Expression Induced by HPP in Macrophages

**Figure 4:**
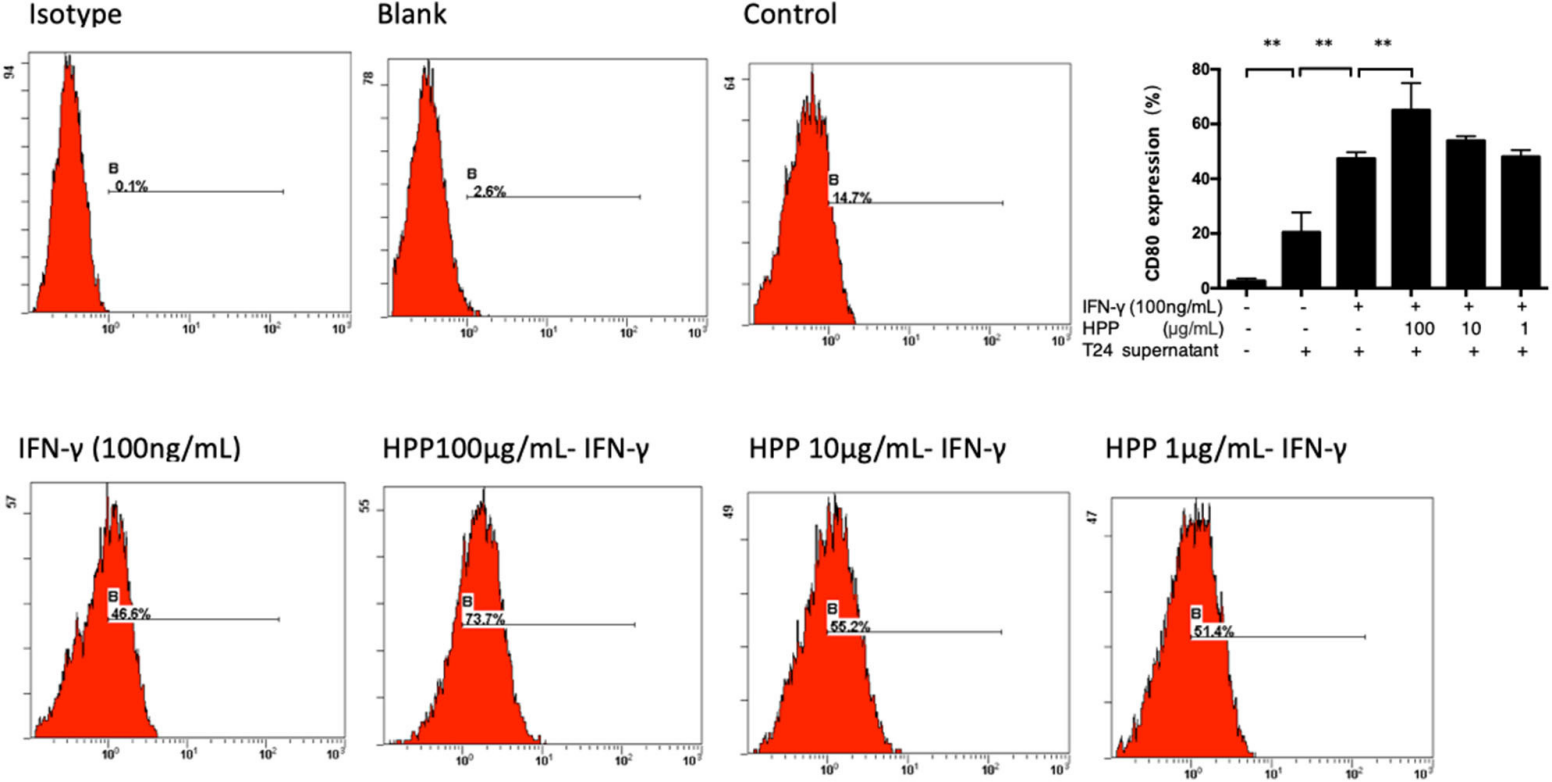
Effect of HPP on CD80 expression of IFN-γ-induced RAW264.7 macrophages. Statistical significance in comparison to the control group is designated *P < 0.05, **P < 0.01. Data are presented as the mean ± s.e.m.

Two *in vitro* effector states (or polarization states) have been widely recognized, namely, M1 and M2 macrophages. M1 macrophages are characterized by the expression of the membrane molecule CD80. TLR-2 signaling augments the expression of the costimulatory molecule CD80 on macrophages and the production of cytokines such as IL-6, IL-12, and TNF-α (Harris et al., 1997; Vidyarthi et al., 2018). In addition, macrophages are important antigen-presenting cells, and their differentiation and activation can activate T cells. Moreover, the accessory function of macrophages depends on the presence of a number of costimulatory CD80 molecules (Meng et al., 2016; Payne et al., 2018). As shown in Figure 4, flow cytometry analysis demonstrated that HPP could increase the signature marker CD80 in IFN-γ-stimulated cells, suggesting that HPP enhanced the activation of RAW 264.7 macrophages.

### HPP Enhanced the Secretion of Pro-inflammatory Cytokines in IFN-γ-Stimulated Macrophages

**Figure 5:**
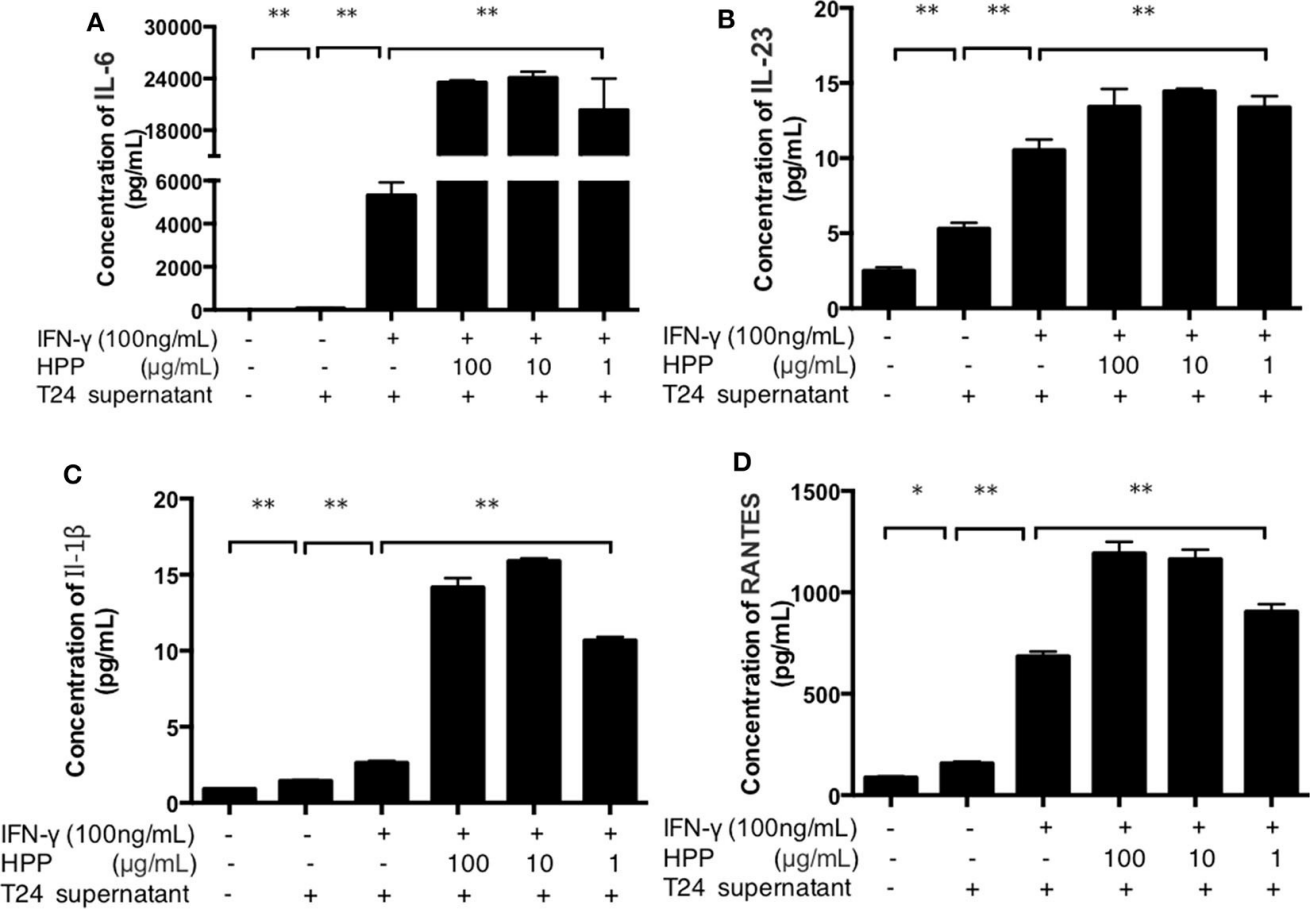
Effect of HPP on the secretion of IL-1β, IL-23, RANTES and IL-6 in IFN-γ-induced macrophages. **(A)** Effect of HPP on IL-6 secretion. **(B)** Effect of HPP on IL-23 secretion. **(C)** Effect of HPP on IL-1β secretion. **(D)** Effect of HPP on RANTES secretion. Statistical significance in comparison to the control group is designated *P < 0.05, **P < 0.01. Data are presented as the mean ± s.e.m.

It is well known that the basic mechanisms of immunomodulators occur via the activation of macrophages, which are secreted as a series of macrophage-derived biological factors (NO, TNF-α, IL-6, IL-1β, IL-10, IL-12, etc.; Wang et al., 2017). IL-23, IL-1β, IL-6 and RANTES are important active molecules that play key roles in the immune system (Michael et al., 2015; Meurette and Mehlen, 2018). When exogenous pathogens, cancer or immunological diseases threaten the host, these molecules are produced by activated macrophages. Taken together, these results indicate that HPP displays notable immunomodulatory activities by increasing the secretion of IL-23, RANTES, IL-6 and TNF-α in RAW264.7 cells. As shown in Figure 5, multiplex immunoassay results showed that the secretion of IL-1β was enhanced in IFN-γ-induced cells treated with HPP (10–100 μg/mL). In addition, when compared with that in the IFN-γ group, the amount of IL-6 increased significantly after treatment with 10 μg/mL HPP (*P* < 0.01). Moreover, HPP had similar concentration-dependent effects on the production of IL-23 and RANTES in IFN-γ-induced RAW264.7 cells. These data suggest that the enhanced inflammation in IFN-γ-stimulated RAW264.7 cells induced by HPP treatment was correlated with expression of pro-inflammatory cytokines.

### The NLRP3 and NF-κB Pathways Participate in Macrophage Activation Induced by HPP

As mentioned above, the range of cytokines (NO, IL-23, IL-1β, IL-6, and RANTES) in RAW264.7 cells induced by HPP raises interesting questions regarding the signaling pathways activated. Several mechanisms involved in cytokine production have been reported, among which upregulation of the NF-κB and NLRP3 pathways has been proposed as a main mechanism (Mishra et al., 2013; Serra et al., 2018). Therefore, we investigated whether HPP could enhance immune-stimulatory activities in the tumor microenvironment through the NF-κB and NLRP3 pathways.

**Figure 6:**
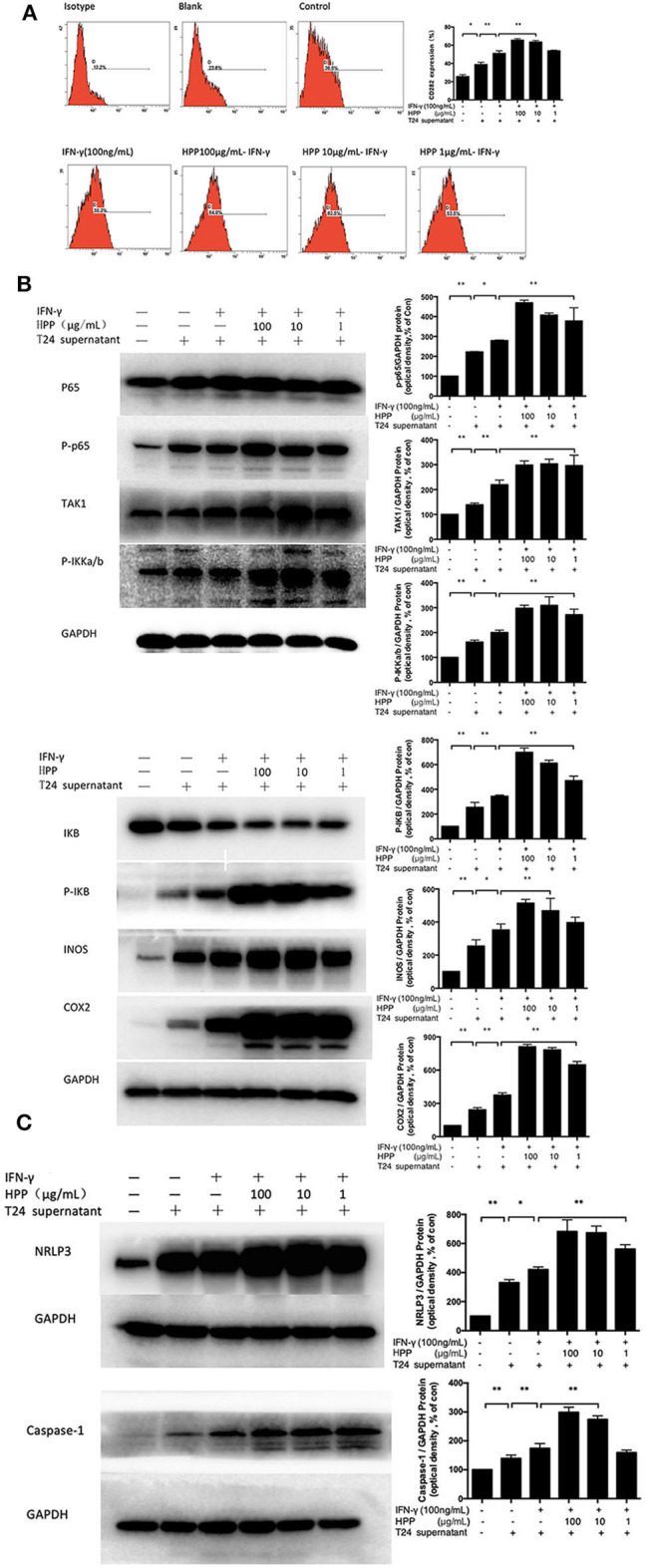
Effect of HPP on the NF-κB/NLRP3 pathway in IFN-γ-induced RAW264.7 macrophages. **(A)** Effect of HPP on the expression of TLR2. **(B)** Effect of HPP on the phosphorylation of NF-κB. **(C)** Effect of HPP on the expression of NLRP3. Statistical significance in comparison to the control group is designated *P < 0.05, **P < 0.01. Data are presented as the mean ± s.e.m.

Pattern recognition receptors (PRRs) have been reported on macrophages that recognize polysaccharides to activate macrophages to participate in the immune regulation process. Studies have shown that polysaccharide from polyporus can bind to TLR2 and activate the downstream NF-κB signaling pathway (Wei, 2009). As shown in Figure 6A, HPP at concentrations of 10–100 μg/mL significantly enhanced the expression of TLR2 (*p* < 0.01). To investigate whether HPP is involved in the activation of macrophages, the expression of TLR2 on cells was determined by flow cytometry.

The NF-κB pathway plays central roles in regulating immune and inflammatory processes that regulate the expression of cytokines involved in immunity and inflammation (Kim et al., 2016). NF-κB complexes are normally held inactive in the cytoplasm by binding to members of the IKB family of proteins in resting cells. In addition, NF-κB can be activated in response to a large variety of stimuli, such as IFN-γ and LPS, causing the phosphorylation of IKB proteins on conserved serine residues by the IKB kinase (IKK) complex (Begalli et al., 2017). HPP was shown to increase the expression of IKKa/b, IKB, TAK1, iNOS, COX2, and NF-κB P65 in RAW264.7 macrophages. Additionally, macrophages activated through the NF-κB pathway produce cytotoxic molecules and inflammatory cytokines, such as NO, IL-6, RANTES, IL-23, and IL-1β (Afonina et al., 2017). To investigate whether the NF-κB signaling pathway participated in IFN-γ-induced macrophage activation by HPP, RAW264.7 cells were pretreated with T24 cell culture supernatant for 3 h and then stimulated with IFN-γ or HPP (1–100 μg/mL) for 3 h. Western blot analysis showed that the expression of P65-NF-κB, TAK1, IKKa/b, P-IKB, INOS, and COX2 increased vitally after treatment with HPP for 8 h (Figure 6B).

The NLRP3 inflammasome is a polyprotein complex of approximately 700 kDa that plays a crucial role in the inflammatory process (Zhang et al., 2012). ASC protein is important for NLRP3 activation. It has been reported that RAW264.7 cells cannot express ASC protein, so NLRP3 cannot be activated in these cells (Pelegrin, 2008). However, in recent years, a large number of literatures have reported that ingredients of Chinese medicine can regulate the ASC of RAW264.7 cells and activate NLRP3 pathway. Pinellia pedatisecta lectin was reported to bind with NLRP3 through ASC following by caspase-1 in the inflammation of RAW264.7 cells (Wang et al., 2019). And luteolin was reported to decrease production of ROS and expression of NLRP3, ASC, caspase-1, IL-18 and IL-1β proteins in RAW264.7 cells, which were incubated with LPS (Zhang et al., 2018). In addition, the Smilax glabra Roxb polysaccharide could evidently promote the phagocytosis and increase macrophage-derived biological factors, including nitric oxide (NO), interleukin-6 (IL-6), and interleukin-1 (IL-1β) secretion, via JNK and ERK signaling pathways and NLRP3 inflammasome signaling pathway (Wang et al., 2017). Our study also found that polyporus polysaccharide can activate NLRP3. This difference may be due to the differential expression of the ASC gene in the different types/sources of RAW264.7 cell lines or various culture condition. NLRP3 inflammasome assembly results in the activation of caspase-1, which in turn processes pro-IL-1β and pro-IL-18 into their mature forms, which initiate inflammation (Afonina et al., 2017; Kaufmann et al., 2017). When NLRP3 is activated, pro-caspase-1 is cleaved, resulting in caspase-1 activation (Zhang et al., 2012). Caspase-1 is essential to the production of mature interleukin-1β (IL-1β) and interleukin-18 (IL-18) in response to a variety of agonists or stimuli. Activated caspase-1 subsequently leads to the processing of the interleukin-1β (IL-1β) and interleukin-18 (IL-18) pro-inflammatory cytokines. Active mature IL-1β is formed by cleavage of the inactive pro–precursor by caspase-1 (Song et al., 2017). IL-1β has been linked to the production of a variety of inflammatory mediators, which play a critical role in the activation and regulation of immune cells and inflammatory responses (Lee et al., 2015; Kaufmann et al., 2017).

To investigate whether HPP is involved in IFN-γ-induced macrophage activation via the NLRP3 signaling pathways, RAW264.7 cells were pre-incubated with T24 cell culture supernatant for 3 h before culturing with IFN-γ for 3 h. The cells were then treated with HPP (1–100 μg/mL) for 8 h, after which western blot analysis was used to detect the expression of NLRP3 and Caspase-1. As shown in Figure 6C, increased NLRP3 expression was observed in cells treated with HPP. Based on the above results, we suggest that HPP-induced increases in the secretion of NO, IL-23, IL-1β, IL-6 and RANTES may have occurred via increases in the NLRP3 and NF-κB pathways.

### Inhibition of the NF-κB Pathways

**Figure 7:**
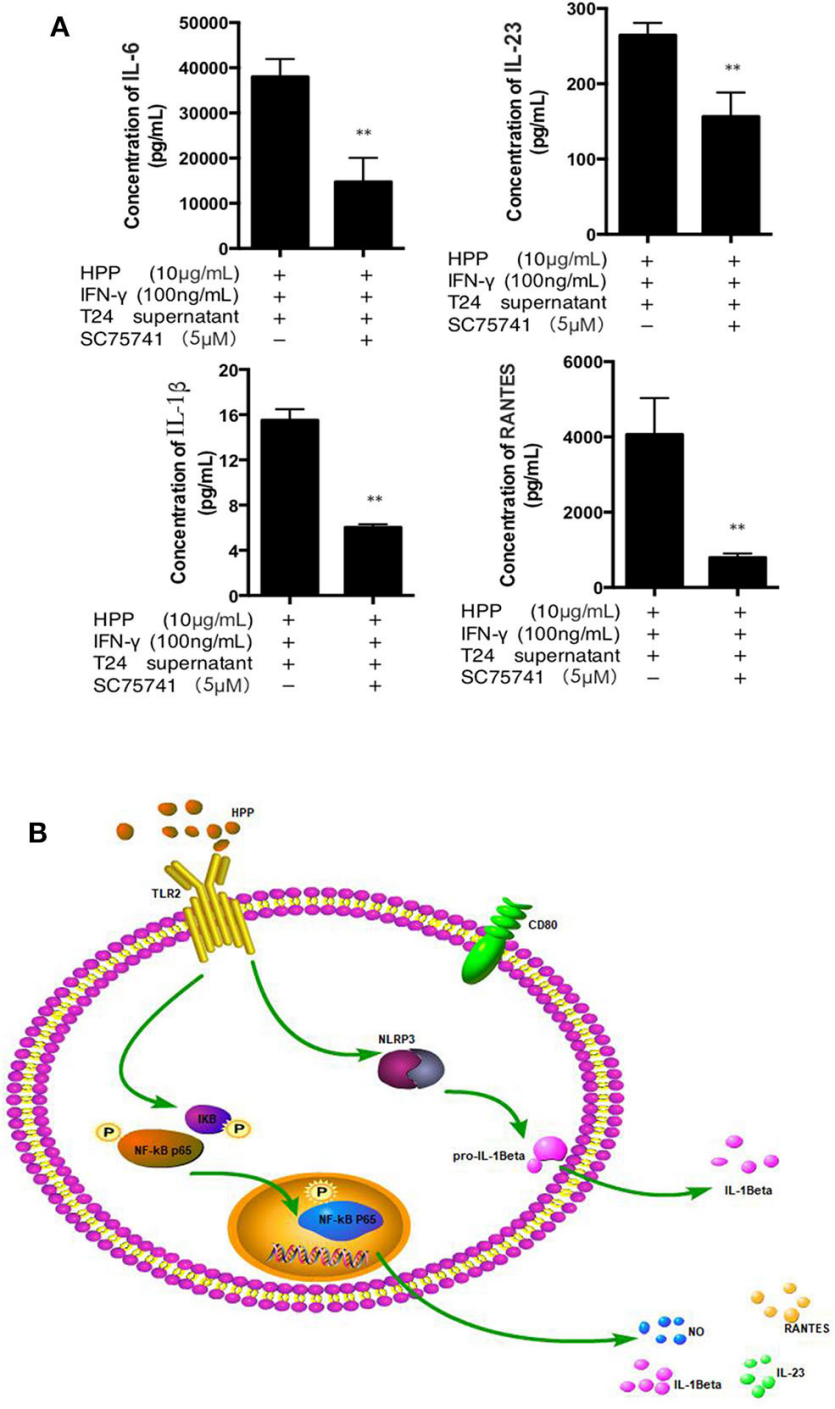
Effect of a specific NF-κB inhibitor on IL-1β, IL-23, RANTES and IL-6 secretion in IFN-γ-stimulated RAW264.7 macrophages. **(A)** Effect of a specific NF-κB inhibitor on IFN-γ-stimulated RAW 264.7 cells treated with HPP. **(B)** Possible molecular mechanisms of HPP-induced macrophage immunomodulation. Statistical significance in comparison to the control group is designated *P < 0.05, **P < 0.01. Data are presented as the mean ± s.e.m.

To further investigate whether the NF-κB signaling pathway was involved in IFN-γ-stimulated macrophage activation induced by HPP, HPP-treated macrophages were exposed to a specific P65-NF-κB inhibitor, SC75741, after which the secretion of IL-1β, IL-23, IL-6 and RANTES was measured using a Procarta PlexTM multiplex immunoassay. As shown in Figure 7A, the NF-κB inhibitor SC75741 attenuated the HPP-induced enhancement of the inflammatory factors IL-1β, IL-23, IL-6 and RANTES by 29, 25.2, 37.5, and 43.8%, respectively, in IFN-γ-stimulated macrophages. As shown in Figure 7B, our results demonstrated that HPP may first bind to the TLR2 receptor in macrophages and that the possible molecular mechanisms of HPP-stimulated macrophage immunomodulation may primarily occur via the NF-κB/NLRP3 signaling pathways, leading to the secretion of IL-1β, IL-23, IL-6, and RANTES.

Taken together, macrophages are the most dynamic and versatile cells involved in steady-state homeostasis, innate immune surveillance, and inflammation establishment and resolution. To elucidate how HPP facilitates the suppression of bladder cancer *in vitro*, it is necessary to investigate the tumor microenvironment. Our studies have reported that RAW 264.7 cells treated with T24 cell culture supernatant may be an ideal macrophage model for vitro studies (Liu et al., 2017). In our present work, we treated RAW 264.7 cells with T24 cell culture supernatant to simulate the bladder tumor microenvironment *in vitro*. In the tumor microenvironment, macrophages that infiltrate into the tumor are generally called TAMs, which are similar to M2 macrophages. We found that HPP enhanced the activities of IFN-γ-stimulated RAW 264.7 macrophages, as shown by the release of NO, IL-6, RANTES, IL-23, and IL-1β, as well as expression of M1 phenotype indicators CD80 via the NF-κB and NLRP3 pathways in the bladder cancer microenvironment. HPP may be bound to TLR2, which is on the surface of macrophages, and a signal was transduced into the cell to participate in the process of activating macrophages. As shown in Figure 7B, the process in macrophages may be as follows: the NF-κB and NLRP3 proteins are stimulated by TLR2 signaling, resulting in activation of the secretion of NO, IL-6, RANTES, IL-1β, and IL-23 and thereby leading to the activation of CD 80.

## Conclusion

In this study, our results present evidence that a new polysaccharide was separated from polyporus. Its monosaccharide composition was glucose, and the main linkage type was proven to be an α-(1 → 4)-linked D-galactan backbone. *In vitro* bioactivity tests showed that HPP has significant immune activity and may alter the tumor microenvironment by regulating the secretion of the inflammatory cytokines NO, IL-6, RANTES, IL-23, and IL-1β. The NF-κB and NLRP3 pathways are thought to be involved in immune regulation by HPP. Therefore, this homogeneous polysaccharide may be a potential candidate for application in bladder cancer therapy.

## Data Availability Statement

All datasets generated for this study are included in the article/supplementary material.

## Author Contributions

Corresponding authors XZe and XL designed the experimental ideas. C-PL the first author, was mainly responsible for the completion of the experiment and the analysis and interpretation of the data. G-NL, J-HL, C-YZ, and W-YJ were mainly responsible for revising the article. Other members, including ML, Q-LT, and X-YZ proofread the content. All authors contributed to the article and approved the submitted version.

## Conflict of Interest

The authors declare that the research was conducted in the absence of any commercial or financial relationships that could be construed as a potential conflict of interest.
